# TE-TSS: an integrated data resource of human and mouse transposable element (TE)-derived transcription start site (TSS)

**DOI:** 10.1093/nar/gkad1048

**Published:** 2023-11-13

**Authors:** Xiaobing Gu, Mingdong Wang, Xiao-Ou Zhang

**Affiliations:** Shanghai Key Laboratory of Maternal and Fetal Medicine, Clinical and Translational Research Center of Shanghai First Maternity and Infant Hospital, Frontier Science Center for Stem Cell Research, School of Life Sciences and Technology, Tongji University, Shanghai 200092, China; Shanghai Key Laboratory of Maternal and Fetal Medicine, Clinical and Translational Research Center of Shanghai First Maternity and Infant Hospital, Frontier Science Center for Stem Cell Research, School of Life Sciences and Technology, Tongji University, Shanghai 200092, China; Shanghai Key Laboratory of Maternal and Fetal Medicine, Clinical and Translational Research Center of Shanghai First Maternity and Infant Hospital, Frontier Science Center for Stem Cell Research, School of Life Sciences and Technology, Tongji University, Shanghai 200092, China

## Abstract

Transposable elements (TEs) are abundant in the genome and serve as crucial regulatory elements. Some TEs function as epigenetically regulated promoters, and these TE-derived transcription start sites (TSSs) play a crucial role in regulating genes associated with specific functions, such as cancer and embryogenesis. However, the lack of an accessible database that systematically gathers TE-derived TSS data is a current research gap. To address this, we established TE-TSS, an integrated data resource of human and mouse TE-derived TSSs (http://xozhanglab.com/TETSS). TE-TSS has compiled 2681 RNA sequencing datasets, spanning various tissues, cell lines and developmental stages. From these, we identified 5768 human TE-derived TSSs and 2797 mouse TE-derived TSSs, with 47% and 38% being experimentally validated, respectively. TE-TSS enables comprehensive exploration of TSS usage in diverse samples, providing insights into tissue-specific gene expression patterns and transcriptional regulatory elements. Furthermore, TE-TSS compares TE-derived TSS regions across 15 mammalian species, enhancing our understanding of their evolutionary and functional aspects. The establishment of TE-TSS facilitates further investigations into the roles of TEs in shaping the transcriptomic landscape and offers valuable resources for comprehending their involvement in diverse biological processes.

## Graphical Abstract

**Graphical Abstract:**
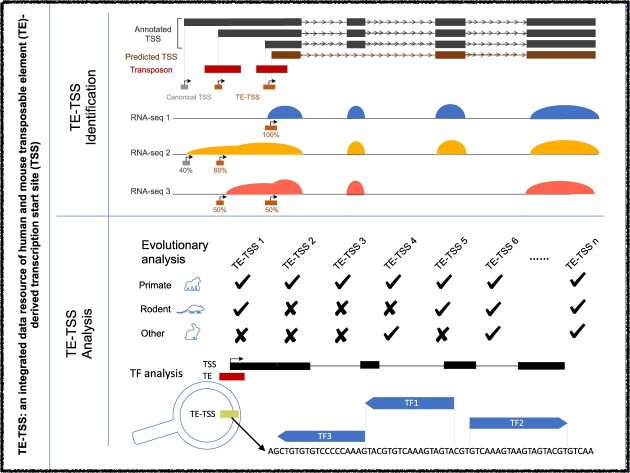

## Introduction

Transposable elements (TEs) are ubiquitous genomic entities that have been acknowledged since the 1950s for their prominent presence within genomes (1). Roughly 45% of the human genome and 37% of the mouse genome consist of TEs (2,3). These TEs can be categorized into retrotransposons and DNA transposons based on their replication mechanism (3–5). Retrotransposons, such as LINEs (long interspersed nuclear elements), SINEs (short interspersed nuclear elements) and LTRs (long terminal repeats), undergo transcription into RNAs followed by reverse transcription into DNA, leading to a progressive amplification of their copy numbers within the genome. Conversely, DNA transposons physically excise themselves from one genomic location and reinsert into another.

Despite the potential threat posed by most TEs to genome stability, necessitating silencing or degenerative mutations to render them inactive (6), certain TEs actively govern genome regulation in diverse biological contexts that include cancer (7,8), immune response (9,10), embryogenesis (11,12) and aging (13,14). Specific TEs function as epigenetically regulated promoters, referred to as TE-derived transcription start sites (TSSs) (11,15). Studies have disclosed that ∼25% of human promoter regions encompass TE-derived sequences (16). Growing lines of evidence underscore the pivotal roles played by TE-derived TSSs in early development (17), specific terminally differentiated tissues (18) and the transcriptional activation of genes involved in immunity or stimulus-response (19). For instance, in lung cancers, an *AluJb* element positioned upstream of the *LIN28B* oncogene operates as an alternative TSS, fostering cancer-specific expression of *LIN28B* isoforms (8). Additionally, during mouse preimplantation development, a mouse-specific *MT2B2* retrotransposon-derived TSS produces an N-terminally truncated Cdk2ap1 protein that peaks in preimplantation embryos, contributing to augmented proliferation (11).

In addition to several established databases cataloging TSSs, such as TRRD (20), FANTOM5 (21), DBTSS (22), refTSS (23) and EPDnew (24), recent studies have collected a wealth of data on TE-derived TSSs. Notably, Shah *et al.* developed a computational tool to systematically identify noncanonical transcripts that originate from TEs and splice into genes (25). Their work extended to the analysis of over 10 000 tumor samples spanning 33 cancer types from The Cancer Genome Atlas datasets, along with 675 cancer cell lines. Similarly, Merlotti *et al.* (26) and Burbage *et al.* (27) have identified hundreds of peptides derived from TE-derived TSSs in human and mouse models, further expanding the repertoire of TE-derived transcripts. However, the lack of an accessible and comprehensive database specifically dedicated to TE-derived TSS data impedes thorough exploration of the intricate TE-mediated transcriptional regulation network.

To bridge this existing gap, we introduce TE-TSS, a comprehensive and carefully curated web resource designed to discern human and mouse TE-derived TSSs, unveiling the regulatory network governed by TE-derived alternative promoters. TE-TSS encompasses 5768 TE-derived TSSs in humans and 2797 in mice, meticulously characterized for their usage across 2681 high-quality biosamples. The user-friendly TE-TSS interface facilitates interactive exploration and visualization of each TE-derived TSS across multiple datasets. With regular updates and maintenance, TE-TSS is poised to furnish valuable insights into the functional significance of TE-mediated regulation and unveil the intricate regulatory networks involving TE-derived TSSs.

## Materials and methods

### Data collection and curation

We systematically compiled human and mouse RNA sequencing (RNA-seq) datasets from several public databases, including the ENCODE project (28) and the Gene Expression Omnibus database (29). Essential meta-information, such as biosample characteristics, sequencing platforms and sequencing types, was systematically gathered and parsed. The collected RNA-seq datasets were further manually checked to remove datasets that exhibited evident 3′ coverage bias or were potentially susceptible to such bias due to the adopted protocols. After filtering to exclude datasets of inadequate quality and those lacking sufficient sample details, the TE-TSS database contains 2681 high-quality RNA-seq datasets, encompassing 1205 human samples and 1476 mouse samples (Supplementary Table S1). Raw RNA-seq reads, acquired from our curated data collection, underwent initial preprocessing using Trimmomatic (version 0.6.10) (30) to remove low-quality sequences and adapter fragments. Next, the resulting clean reads were aligned against the hg38/GRCh38 reference genome for human samples and against the mm39/GRCm39 reference genome for mouse samples using STAR (version 2.7.10b) (31) with the two-pass alignment mode (parameter: ‘--twopassMode Basic’). Employing this optimized alignment strategy facilitated the identification of novel splicing junctions, consequently enhancing the precision of promoter usage quantification in downstream analyses. For accurate annotation of expressed TSSs, we combined TSS annotations from the human curated TSS collection (representative peaks) (32), which were established through the integration of high-quality ENCODE RAMPAGE (RNA annotation and mapping of promoters for the analysis of gene expression) experiments (33), and the human and mouse promoter expression atlas using FANTOM5 CAGE (cap analysis gene expression) datasets (34). Additionally, available data from GRO-cap (35), PRO-cap (36), csRNA-seq (37) and NET-CAGE (38) were also included to annotate TSSs.

### Identification of expressed TE-derived TSSs and estimation of promoter usage

**Figure 1.**
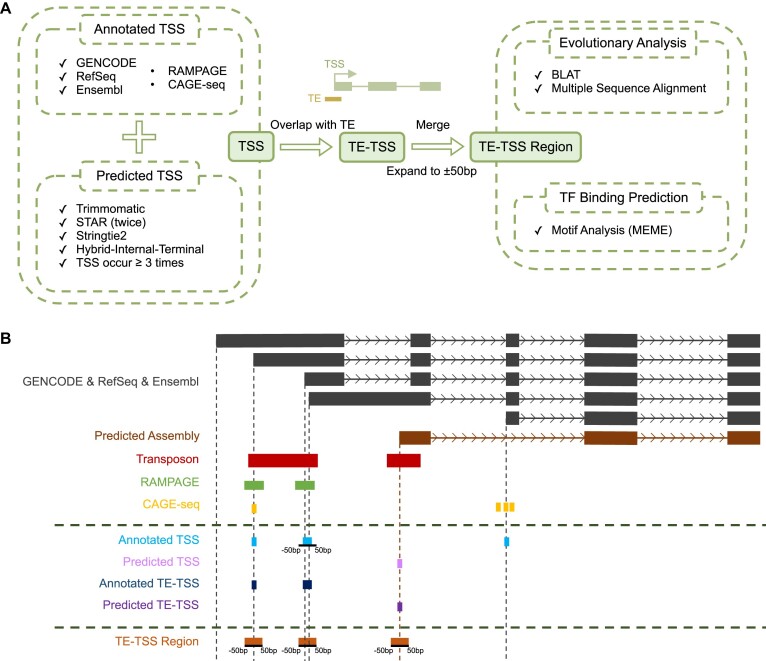
TE-TSS workflow. (**A**) The TE-TSS pipeline involves the integration of annotated and predicted TSSs. TE-derived TSSs were identified by intersecting TEs with TSSs, followed by the merging of adjacent TE-derived TSSs. The resulting regions were then expanded by ±50 bp to establish TE-derived TSS regions, which formed the basis for subsequent evolutionary and functional analyses. (**B**) Schematic diagram showing the identification of annotated/predicted TSSs and TE-derived TSSs.

**Figure 2.**
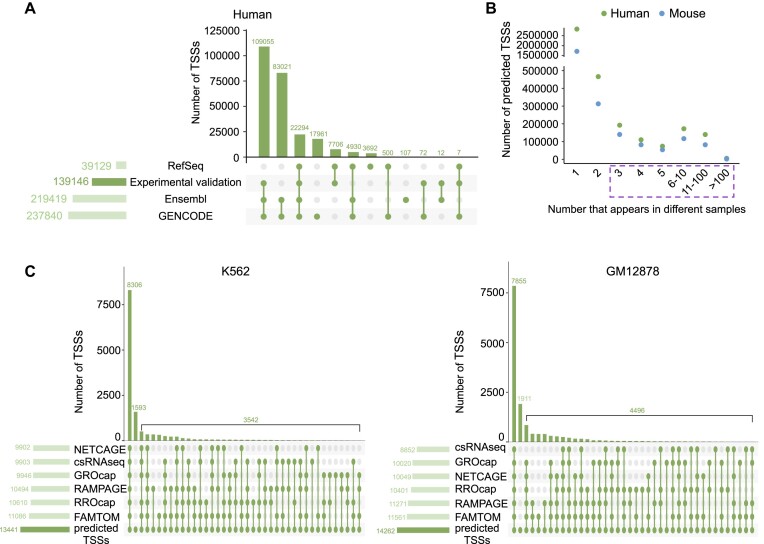
Integration and analysis of human and mouse TSS references for usage estimation. (**A**) Upset plot displaying the distribution of human TSSs across RefSeq, GENCODE and Ensembl, as well as TSSs among them that have experimental validation by existing TSS assays. These TSSs with experimental validation are defined as annotated TSSs. (**B**) Distribution of TSSs identified across varying numbers of samples. Predicted TSSs were retained if they appeared in a minimum of three samples. (**C**) Upset plots showing the distribution of predicted TSSs overlapping with existing TSS assays in K562 (left panel) and GM12878 (right panel) cells.

The TE-TSS database comprises both annotated TSSs and predicted TSSs. Annotated TSSs were compiled through the integration of TSS annotations from RefSeq (update on 11 April 2023) (39), GENCODE (v44 for human, vM33 for mouse) (40) and Ensembl (Release 109) (41) databases, and were further experimentally validated based on their overlap with other available TSS assays, including RAMPAGE or CAGE-seq peaks. To predict novel TSSs from individual RNA-seq samples, a two-step strategy was employed. First, transcriptome assembly was conducted using StringTie2 (42) to reconstruct putative transcripts. Subsequently, the hybrid–internal–terminal (HIT) pipeline (43) was employed to predict novel TSSs from each distinct RNA-seq dataset. The HIT pipeline utilizes probabilistic modeling ratios of splice junction coverage to identify first or hybrid first–internal exons. TSSs of reconstructed transcripts that overlapped with first or hybrid first–internal exons were defined as the predicted TSSs. To ensure the reliability of predicted TSSs, we applied a stringent criterion, retaining only those TSSs predicted in at least three different samples (Figures 1A and 2B).

Annotated TE-derived TSSs and predicted TE-derived TSSs were further identified from the annotated and predicted TSSs, respectively, through overlapping with TE annotations from the UCSC RepeatMasker database (44). To eliminate potential TE-only transcripts, assembled transcripts consisting of only one exon or those with a length of <200 bp were excluded. In addition, we removed all TE-derived TSSs that could not be assigned to any annotated genes. To address the close proximity of many TSSs, we merged TSSs within ±50 bp into a single TSS. To explore the evolutionary and functional characteristics of the TE-derived TSSs, we extended the sequence by ±50 bp around the TE-derived TSSs, defining them as the TE-derived TSS region for further analysis (Figure 1B).

To estimate the usage of TSS in each biosample, we adopted the percent spliced in (PSI) values specifically calculated for alternative first exons using the HIT pipeline. Each TSS is assigned a PSI value ranging from 0 to 1, representing its usage level within the expressed genes.

### Analysis of TE-derived TSS conservation in multiple species

To unravel the conservation of TE-derived TSS regions across various species (Figure 1A), we employed BLAT (45) for pairwise sequence alignment. For human TE-derived TSS regions, we conducted BLAT analysis on the following primate species: baboon (papAnu4), chimpanzee (panTro6), gibbon (nomLeu3), rhesus (theMac10), crab-eating macaque (macFas5) and green monkey (chlSab2); as well as two rodent species: rat (rn7) and mouse (mm39). For mouse TE-derived TSS regions, we performed BLAT analysis on two rodent species: rat (rn7) and Chinese hamster (criGriChoV2); three primate species: human (hg38), chimpanzee (panTro6) and rhesus (theMac10); and pika (ochPri3). Additionally, we aligned human and mouse TE-derived TSS regions in four other mammalian species: pig (susScr11), dog (canFam4), sheep (oviAri4) and rabbit (oryCun2). To ensure the accuracy of BLAT alignments, we selectively retained sequences based on the BLAT score (38) with minor modifications. The modified BLAT score was calculated using the following equation:


\begin{eqnarray*} && {\rm modified}\ {\rm BLAT}\ {\rm score} \nonumber\\ && \quad =\frac{{{\rm matches} + {\rm repMatches} - {\rm mismatches} - {\rm qNumInsert} - {\rm tNumInsert}}}{{{\rm qSize}}},\end{eqnarray*}

where matches represent the number of bases that match and are not part of repeats, repMatches represent the number of bases that match but are part of repeats, mismatches represent the number of bases that do not match, qNumInsert represents the number of inserts in the query, tNumInsert represents the number of inserts in the target and qSize represents the query sequence size. Sequences with a modified BLAT score >0.7 were retained for further analysis. Additionally, to prevent excessively long insertions in the target sequences, we constrained the target sequence size to be within 80–120% of the query sequence size. Multiple sequence alignment of TE-derived TSS regions across diverse species was performed using pyMSAviz (pypi.org/project/pymsaviz/).

### Motif analysis of TE-derived TSS regions

To identify potential transcription factor (TF) binding sites in TE-derived TSS regions, we conducted motif analysis by scanning each annotated TF motif from the JASPAR (JASPAR2022 CORE redundant v2) database (46) using the FIMO tool from the MEME suite (47) (Figure 1A). A motif hit with a FIMO *P*-value <10^−4^ was considered a significant predicted TF binding site.

### Tissue specificity of TE-derived TSSs

We evaluated the tissue specificity of TE-derived TSSs with a previously proposed index (48), which varies between 0 for housekeeping genes and 1 for tissue-restricted genes. The gene set of housekeeping genes was fetched from the HRT Atlas database (49).

### Construction of the TE-TSS web interface

The TE-TSS database is hosted within a stable Nginx environment (v1.21.6) on a CentOS 7 Linux system, providing reliable web access. All datasets were processed and stored in a MySQL database system (version 14.14). For seamless user experience, we implemented the database query and user interface using Django, Layui, AJAX and Vue.js. To enhance data visualization, interactive plots were generated using the Plotly JavaScript library (https://plotly.com/javascript/). For the visualization of TE-derived TSS annotation and usage across different biosamples, we employed JBrowse2 (50), a web-based genome browser.

## Results

### Integration and analysis of human and mouse TSS references for usage estimation

Two prominent challenges in TSS research arise from the limited experimental validation of current TSS annotations and the absence of cell type-specific usage information for each TSS. Techniques such as CAGE and RAMPAGE, designed to capture and preserve 5′ ends, are constrained by limited biosamples. To address these issues, our strategy centered on establishing a comprehensive human and mouse TSS reference. We aggregated TSS annotations from reputable databases, including RefSeq, GENCODE and Ensembl, retaining those substantiated by experimental validation through available TSS assays. This compilation yielded 139 146 annotated TSSs for humans and 79 616 for mice (Figure 2A and Supplementary Figure S1A). Subsequently, by employing the HIT pipeline (43), we predicted expressed TSSs directly from individual RNA-seq datasets that exhibited no coverage bias (Supplementary Figure S1B), filtering for occurrences in a minimum of three distinct samples. This approach led to the identification of 693 147 predicted TSSs in humans and 475 456 in mice (Figure 2B). As core promoter generally spans a 100 bp region (51), we consolidated proximal TSSs within this range into single entities, resulting in 227 679 TSS candidates (70.8% predicted) in humans and 143 324 (70.7% predicted) for mice (Supplementary Table S2). To evaluate the accuracy of predicted TSSs, we checked the percentage of predicted TSSs that were corroborated by alternative TSS assays conducted within the same samples. Our results revealed that 88% and 87% of the predicted TSSs in K562 and GM12878, respectively, were consistently identifiable by at least one of these well-established TSS assays (Figure 2C).

Using the capabilities of the HIT pipeline, we can estimate TSS usage and perform cross-TSS comparisons. For example, our pipeline successfully predicted novel TSSs in *ATP2B4* and BTBD16 within the liver tissue (Supplementary Figure S1C). Notably, these predicted TSSs function as the predominant TSSs in their respective genes, exhibiting a higher TSS usage when compared to annotated TSSs. Furthermore, these findings were corroborated by RAMPAGE data obtained from the same biosample (Supplementary Figure S1C).

In summary, our TSS annotation strategy effectively addressed these challenges by integrating existing experimental data with advanced computational predictions to establish a comprehensive TSS reference. The employment of the HIT pipeline enabled both TSS identification and exploration of dynamic TSS usage patterns across distinct contexts.

### Construction of the TE-derived TSS atlas

**Figure 3.**
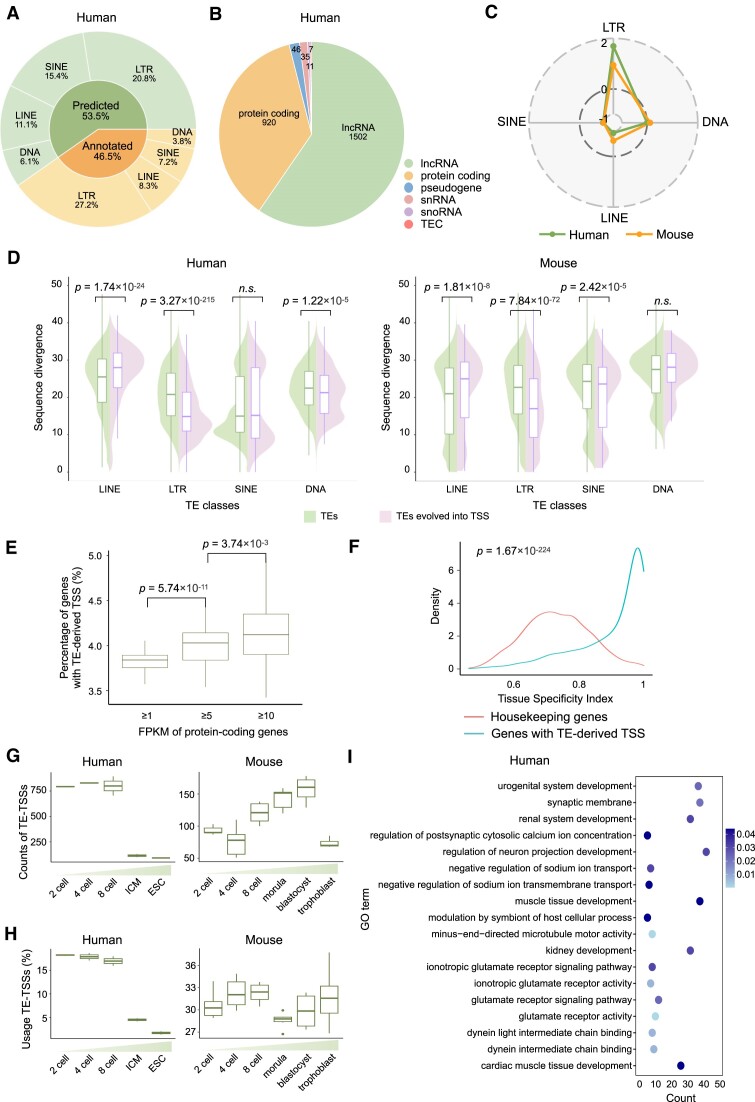
Identification of TE-derived TSSs. (**A**) Composition of predicted and annotated TE-derived TSSs, along with the distribution of TE classes in the human genome, depicted in a pie chart. (**B**) Distribution of gene types associated with human annotated TE-derived TSSs, categorizing 1502 lncRNAs, 920 protein-coding genes and 46 pseudogenes. (**C**) Radar plot illustrating the enrichment score of TE-derived TSSs originating from distinct TE classes. The enrichment score, representing the log_2_ ratio of observed counts to expected counts, is indicative of the propensity for TEs within each class to evolve into TSSs. Observed counts denote the actual number of TEs within the TE class that have evolved into TSSs, while expected counts are calculated under the assumption of equal probability for TEs from each class to evolve into TSSs. (**D**) Beanplot showing the distribution of sequence divergence for different TE classes, including those TEs that have evolved into TSSs. The presentation includes boxplots depicting the median and interquartile range (IQR), with whiskers extending to 1.5 times the IQR. Wilcoxon rank-sum test *P*-values are shown. (**E**) The protein-coding genes with higher expression levels are more likely to have TE-derived TSSs. Wilcoxon rank-sum test *P*-values are shown. (**F**) Genes with TE-derived TSSs exhibit significantly higher (*P*-values <2.2 × 10^−16^, Wilcoxon rank-sum test) tissue specificity than housekeeping genes. (**G**) Identification of TE-derived TSSs in different human and mouse embryonic cells, depicted through boxplots illustrating the median and IQR, with whiskers extending to 1.5 times the IQR. (**H**) Usage of TE-derived TSSs in different human and mouse embryonic cells. Boxplots display median and IQR, with whiskers extending to 1.5 times the IQR. (**I**) Gene ontology analysis of genes with tissue-specific TE-derived TSSs detected in human embryonic cells.

By intersecting with TE annotations from the UCSC RepeatMasker database, we successfully identified 5768 TE-derived TSSs in humans and 2797 in mice (Supplementary Table S3). Among these, 53% of human TE-derived TSSs and 62% of mouse TE-derived TSSs were predicted (Figure 3A and Supplementary Figure S2A). These TE-derived TSSs govern diverse downstream genes, encompassing 1502 long noncoding RNAs (lncRNAs), 920 protein-coding genes and 46 pseudogenes in humans, as well as 550 lncRNAs and 395 protein-coding genes in mice (Figure 3B and Supplementary Figure S2B). Compared to canonical TSSs, a significantly higher concentration of TEs was observed in the immediate vicinity of TE-derived TSS regions (Supplementary Figure S2C). Similar to canonical TSSs, TE-derived TSSs exhibited elevated levels of H3K4me3, H3K9ac, H3K27ac, H3K4me2 and H3K4me1 in corresponding cell lines, whereas no significant increase in these epigenetic marks was observed at randomly selected TEs (Supplementary Figure S2D).

Notably, specific TE classes exhibit preferences for TSS generation. LTRs stand out as the most abundant transposon class contributing to TE-derived TSSs, accounting for 58% of annotated human TE-derived TSSs, 39% of predicted human TE-derived TSSs, 61% of annotated mouse TE-derived TSSs and 40% of predicted mouse TE-derived TSSs (Figure 3A and Supplementary Figure S2A). Enrichment analysis highlights the pronounced propensity of LTRs to evolve into TSSs (Figure 3C and Supplementary Figure S2E). Within the LTR class, the *Gypsy* and *ERVL* families in mice, as well as the *ERV1* and *ERVK* families in humans, demonstrated a higher prevalence in evolving into TE-derived TSSs (Supplementary Figure S2F). Moreover, our investigation into the relationship between TE evolutionary age and TSS emergence reveals a striking trend: young LTRs and old LINEs are particularly inclined to evolve into TSSs, as discerned from sequence divergence comparisons (Figure 3D). Upon inspecting TE families, it was revealed that old *L1* elements and young *MaLR*, *ERV1*, *ERVL* and *Alu*/*B1* elements are more prone to evolving into TSSs (Supplementary Figure S2G).

It is worth noting that TE-TSS genes exhibited distinct expression patterns and might be involved in significant biological processes. For instance, in-depth analysis using the EN-TEx dataset, which comprises RNA-seq data from 91 datasets across 29 tissue types from four human donors, revealed that TE-TSS genes exhibit significant enrichment among highly expressed genes (Figure 3E) and display greater tissue specificity when compared to housekeeping genes (Figure 3F). During embryonic development (52,53), TE-derived TSSs exhibited an initial increase followed by a subsequent decrease (Figure 3G). The proportion of genes utilizing TE as an alternative TSS diminished as embryogenesis progressed (Figure 3H). Gene ontology analysis on genes with TE-derived TSSs during human embryonic development revealed significant enrichments in terms related to human embryonic development, such as neuron projection development and muscle tissue development (Figure 3I), suggesting that TE-TSS genes may play a crucial role in the developmental processes.

### Evolutionary analysis of TE-derived TSS regions

To comprehensively assess the evolutionary conservation of TE-derived TSS regions, we conducted BLAT alignments to compare these regions across diverse species, aiming to unveil the extent of sequence homology and conservation within varying evolutionary contexts. The sequence similarity was quantified using the modified BLAT score, revealing a positive correlation between species evolutionary proximity and higher modified BLAT scores (Supplementary Figure S3A). Remarkably, 97.4% of human TE-derived TSS regions exhibited homology with those in chimpanzees, yielding BLAT scores exceeding 0.8. Similarly, 91.8% of sequences displayed homology and BLAT scores surpassing 0.7 in other primate species. The primate-specific *Alu* element demonstrated heightened conservation in primate species, signifying an increased BLAT score for human *Alu*-derived TSS regions compared to non-primate species (Supplementary Figure S3B). Conversely, the mammalian interspersed repeats (*MIR*s), an ancient TE class pervasive among mammals, exhibited remarkable conservation for *MIR*-derived TSS regions across diverse mammalian species (Supplementary Figure S3C).

**Figure 4.**
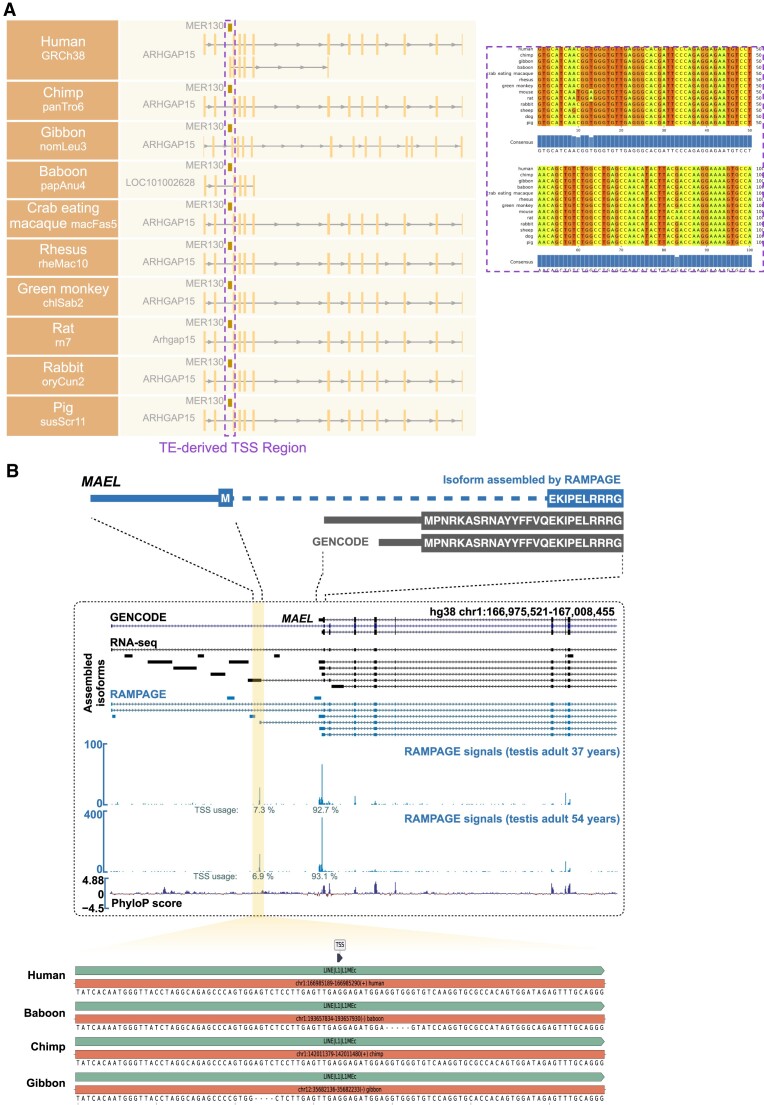
Evolutionary analysis of TE-derived TSSs. (**A**) (Left) Schematic diagram of the gene structure of *ARHGAP15* with a *MER130*-derived alternative TSS across various species. The dotted box highlights the specific region corresponding to the TE-derived TSS. (Right) Multiple sequence alignment of this *MER130*-derived TSS region across various species. (**B**) Schematic diagram showing a primate-conserved *L1MEc*-derived alternative TSS drives the production of a truncated *MAEL* isoform in testis samples.

Among the intriguing observations within these TE-derived TSS regions, we encountered instances where specific TE-derived TSSs were associated with conserved genes that span multiple species. For example, *ARHGAP15*, a regulator of Rho GTPase family members (Rho, Rac and Cdc42) within cells (54), exhibited alternative TSSs in humans, with a downstream TSS originating from the *MER130* element (Figure 4A). This *MER130* element exhibits conservation in other primates and mammals, being positioned downstream of the canonical promoter of *ARHGAP15* (Figure 4A). This occurrence of the conserved *MER130* element raises intriguing possibilities regarding alternative TSS utilization within *ARHGAP15* across disparate species. Another example highlights a primate-conserved *L1MEc* element that can serve as an alternative TSS for the *MAEL* gene in testis tissues (Figure 4B). This TE-derived TSS has the potential to modify the amino acid sequence of the original gene, ultimately resulting in a truncated peptide (Figure 4B).

In summary, our comprehensive analysis of TE-derived TSS region conservation underscores intriguing patterns of sequence homology and preservation across species, shedding light on potential mechanisms of evolutionary adaptation and functional diversity.

### TF binding motif prediction of TE-derived TSS regions

**Figure 5.**
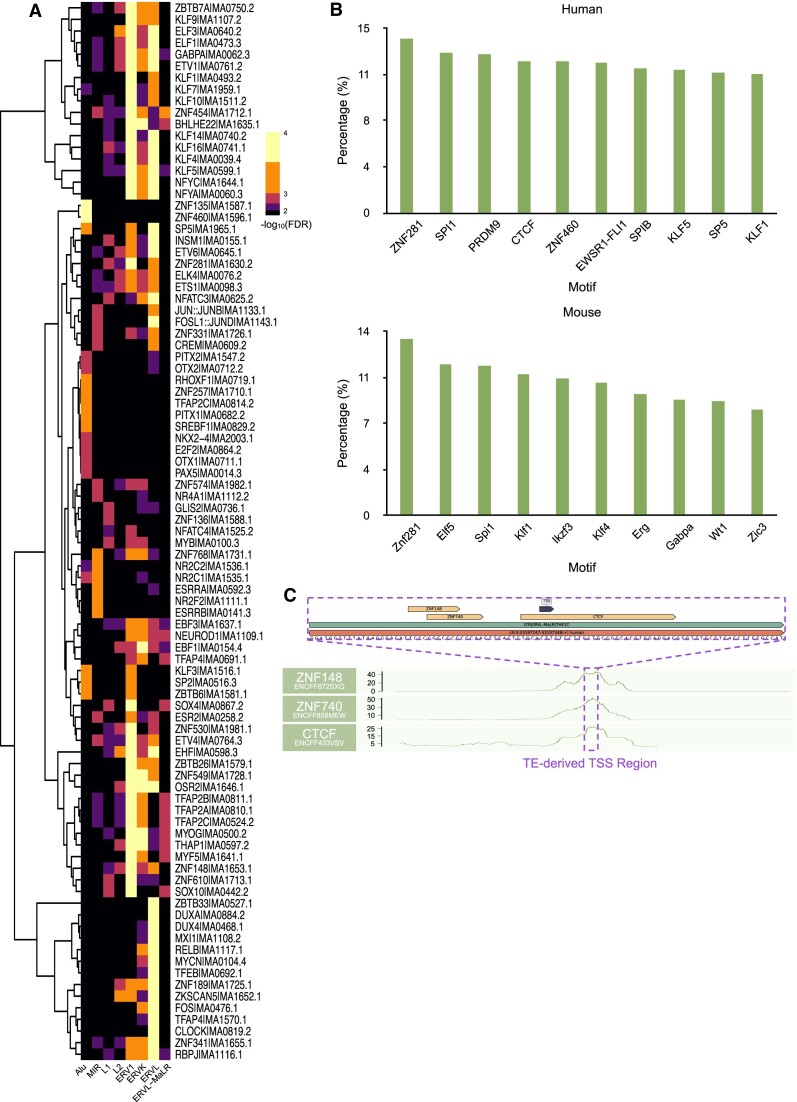
TF binding motifs in TE-derived TSS regions. (**A**) TF motif enrichment at TE-derived TSSs for each TE family. (**B**) Top 10 motifs with the highest occurrence frequency identified within TE-derived TSS regions in both humans and mice. Motifs with *P*-value <10^−4^ are shown. (**C**) An example of an LTR-derived TSS region predicted to contain binding motifs for ZNF148, ZNF740 and CTCF. Notably, ChIP-seq signals for ZNF148, ZNF740 and CTCF exhibit significant peaks in this LTR-derived TSS region.

To gain deeper insights into the regulatory landscape of TE-derived TSS regions, we conducted TF binding motif prediction for these TE-derived TSSs. Notably, compared with randomly selected TEs that do not function as TSSs, distinct human TE families display specific TF enrichment patterns, with a noteworthy emphasis on the prevalence of enriched TFs within LTRs (Figure 5A). The top enriched motifs within human TE-derived TSS regions encompass ZNF281, SPI1 and PRDM9, while in the mouse counterpart, these motifs consist of Znf281, Elf5 and Spi1 (Figure 5B). It is worth noting that many of these predicted motifs found within TE-derived TSS regions exhibited congruence with ChIP-seq data. For instance, an LTR-derived TSS was predicted to harbor motifs for ZNF148, ZNF740 and CTCF. Remarkably, the ChIP-seq signals of these three TFs exhibited pronounced peaks within this specific region (Figure 5C). This convergence between motif prediction and empirical ChIP-seq data provides compelling evidence of the regulatory influence of these TFs on the identified TE-derived TSSs.

### Navigating TE-derived TSS information with TE-TSS database

The TE-TSS database stands as a comprehensive, user-friendly and visually informative resource dedicated to TE-derived TSS information within the human and mouse genomes. TE-TSS encapsulates several core functionalities, collectively empowering researchers to explore and comprehend the multifaceted landscape of TE-derived TSSs.

**Figure 6.**
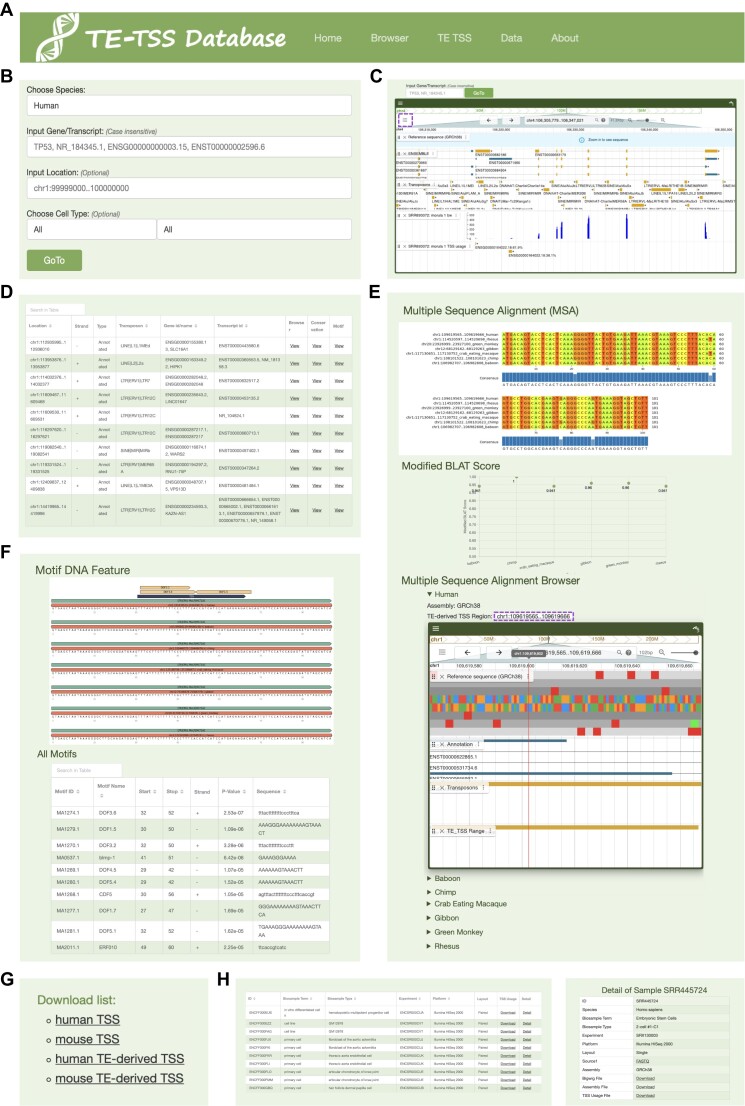
Overview of TE-TSS interface and usage. (**A**) TE-TSS offers five distinctive functional modules. (**B**) Users can search for specific TE-derived TSSs based on species, gene and sample parameters in the Home module. (**C**) The Browser module allows exploration of TSS usage across various samples using the genome browser. The button within the dotted box facilitates switching between sample files. (**D**) The TE-derived TSS search module enables a comprehensive exploration of available features. (**E**) Homology analysis involves multiple sequence alignment, modified BLAT score and consensus sequence browser for TE-derived TSS regions. (**F**) The results of motif analysis are visually presented for the TE-derived TSS region. (**G**) A list of TSSs and TE-derived TSSs is available for download in the Home module. (**H**) Convenient access to RNA-seq file information and associated downloads.

*User-friendly interface*: TE-TSS is structured into five distinct functional modules, comprising Home, Browser, TE TSS, Data and About (Figure 6A). This intuitive layout ensures smooth navigation and easy accessibility.

*TSS usage search*: Within the Home module, users can select their desired species (human/mouse), input specific genes/transcripts or locations of interest, and choose from diverse cell types (Figure 6B). Subsequent interaction with the ‘GoTo’ button seamlessly transitions users to the Browser module (Figure 6C). Here, the genome browser furnishes essential genomic tracks, encompassing genome annotations, TE annotations, RNA-seq signals and TSS usage. The TSS usage depicts TSSs at precise locations, along with their associated genes and utilization proportions within those genes. Notably, Figure 6C exemplifies alternative TSSs, further enhanced by the option to switch displayed tracks via the upper left corner button (dotted purple box). Users can navigate to different regions via the gene/transcript search box and location input box.

*TE-derived TSS search*: Within the TE TSS module, users can effectively explore specific TE-derived TSSs of interest (Figure 6D). This module offers comprehensive details about associated genes and transcripts for each identified TSS. By selecting the ‘View’ option beneath the ‘Browser’ column, users can seamlessly transition to the genome browser, presenting the precise TE-derived TSS region. This immersive view allows users to delve into the expression patterns of the TE-derived TSS across diverse samples (Figure 6C). Furthermore, clicking the ‘View’ option below the ‘Conservation’ column reveals the region’s evolutionary conservation across different species. Users gain access to multiple sequence alignment outcomes, modified BLAT scores and a comprehensive genome browser showcasing homologous regions across varied species (Figure 6E). The genome browser’s intuitive interface permits seamless exploration of homologous regions, while species-specific browsing is facilitated through the triangular arrow on the left side. Additional insights await users via the ‘View’ option below the ‘Motif’ column, which grants access to DNA feature plots of motifs within the TE-derived TSS region (Figure 6F).

*TSS list and data download*: You can download the TSS and TE-derived TSS lists from both the Home module and the TE TSS module (Figure 6G). Meanwhile, access to RNA-seq data employed in TE-TSS is conveniently offered within the Data module (Figure 6H). TE-TSS extends links to raw RNA-seq sample information, bigwig files, assembly files (in bed12.gz format) and TSS usage files (in bed.gz format).

## Discussion

TE-TSS stands out with two distinct features: (i) It incorporates both experimentally validated and computationally predicted TSS data. Experimentally validated TSSs are obtained from known gene annotations and further confirmed through available TSS assays. For computationally predicted TSSs, we employed the HIT pipeline (43), which utilizes a generative modeling method to predict TSSs directly from widely available RNA-seq datasets. A stringent threshold is applied to retain only those TSSs predicted in at least three different samples, ensuring higher accuracy and robustness. Furthermore, TE-TSS predicts the usage proportions of different TSSs within the same gene, providing valuable insights into the study of alternative promoters. (ii) TE-TSS is the first database dedicated specifically to TE-derived TSSs, encompassing 5768 human TE-derived TSSs and 2797 mouse TE-derived TSSs, of which 47% and 38%, respectively, have been experimentally validated, while the rest were strictly predicted. In addition, TE-TSS analyzes the evolutionary aspects and TF binding motifs of TE-derived TSS regions across 15 mammalian species, including 7 primate species, 3 rodent species and 5 other mammalian species.

While TE-TSS offers a comprehensive data collection, there are still avenues for optimization and further development. Future versions of this database will be expanded to include more RNA-seq datasets from various disease conditions as well as healthy controls. With the ongoing advancements in full-length long-read RNA-seq data, TE-TSS will be expanded to incorporate publicly available TE-derived TSSs from long-read RNA-seq. These updates will enhance the value of TE-TSS and broaden its applications in diverse research areas.

In summary, TE-TSS overcomes the limitations of insufficient promoter sequencing data for specific cell types, significantly enriches the information on TSSs across diverse cells, and mitigates biases arising from technical variations. Moreover, it sheds light on the TF binding and evolutionary patterns of TE-derived TSSs, providing valuable insights into their potential functional significance and unraveling the complex regulatory networks involving TE-derived TSSs.

## Supplementary Material

gkad1048_Supplemental_Files

## Data Availability

The TE-derived TSS annotation, differential promoter usage and sample meta-information in the TE-TSS database could be directly downloaded from http://xozhanglab.com/TETSS.
