# Supplementary material for: TE-TSS: an integrated data resource of human and mouse transposable element (TE)-derived transcription start site (TSS)
Source: Nucleic Acids Res. 2023 Nov 13;52(D1):D322–33. doi: 10.1093/nar/gkad1048 (PMC10767810; doi:10.1093/nar/gkad1048)
Supplement: gkad1048_Supplemental_Files [file gkad1048_supplemental_files.zip › Supplementary Figure.pdf]

LEGENDS OF SUPPLEMENTARY FIGURES

Figure S1. An overview of TSS identification and TSS usage. (**A**) Upset plot displaying the distribution of mouse TSSs across RefSeq, GENCODE, and Ensembl, as well as TSSs among them that have experimental validation by existing TSS assays. (**B**) Distribution of read coverage for RNA-seq datasets including in the TE-TSS database. (**C**) Novel TSSs identified in the TE-TSS database serve as the predominant TSSs for *ATP2B4* and *BTBD16* in liver samples.

Figure S2. Overview of TE-derived TSSs. (**A**) Proportions of predicted or annotated TE-derived TSSs and TE classes in mouse shown in a pie chart. (**B**) Distribution of mouse annotated TE-derived TSSs by gene type, including 550 lncRNAs and 395 protein-coding genes. (**C**) TEs are significantly enriched in TE-TSS regions but are absent in canonical TSS regions in humans and mice. (**D**) Average signal of ChIP-seq signals of H3K4me3, H3K9ac, H3K27ac, H3K4me2, H3K4me1, and H3K36me3 per cell type in the ±1-kb regions centered on the TE-derived TSSs, canonical TSSs, and randomly selected TEs in GM12878 and K562 cells. (**E**) Enrichment score of annotated or predicted TE-derived TSSs from various TE classes depicted in a radar plot. (**F**) Radar plot illustrating the enrichment score of TE-derived TSSs originating from distinct LTR families. (**G**) Beanplot showing the distribution of sequence divergence for different TE families, including those TEs that have evolved into TSSs. Wilcoxon rank-sum test p-values are shown.

Figure S3. Modified BLAT Scores of TE-derived TSS Regions. (**A**) Density distribution map illustrating the modified BLAT score distribution of TE-derived TSS regions from human or mouse aligned with different species. (**B**) Distribution of modified BLAT scores for human *Alu*-derived TSS regions across diverse mammals presented in a boxplot. Median and IQR are represented, and whiskers extend to 1.5 times the IQR. (**C**) Boxplot depicting the distribution of modified BLAT scores for human *MIR*-derived TSS regions across various mammals. Median and IQR are represented, and whiskers extend to 1.5 times the IQR.

Figure S1

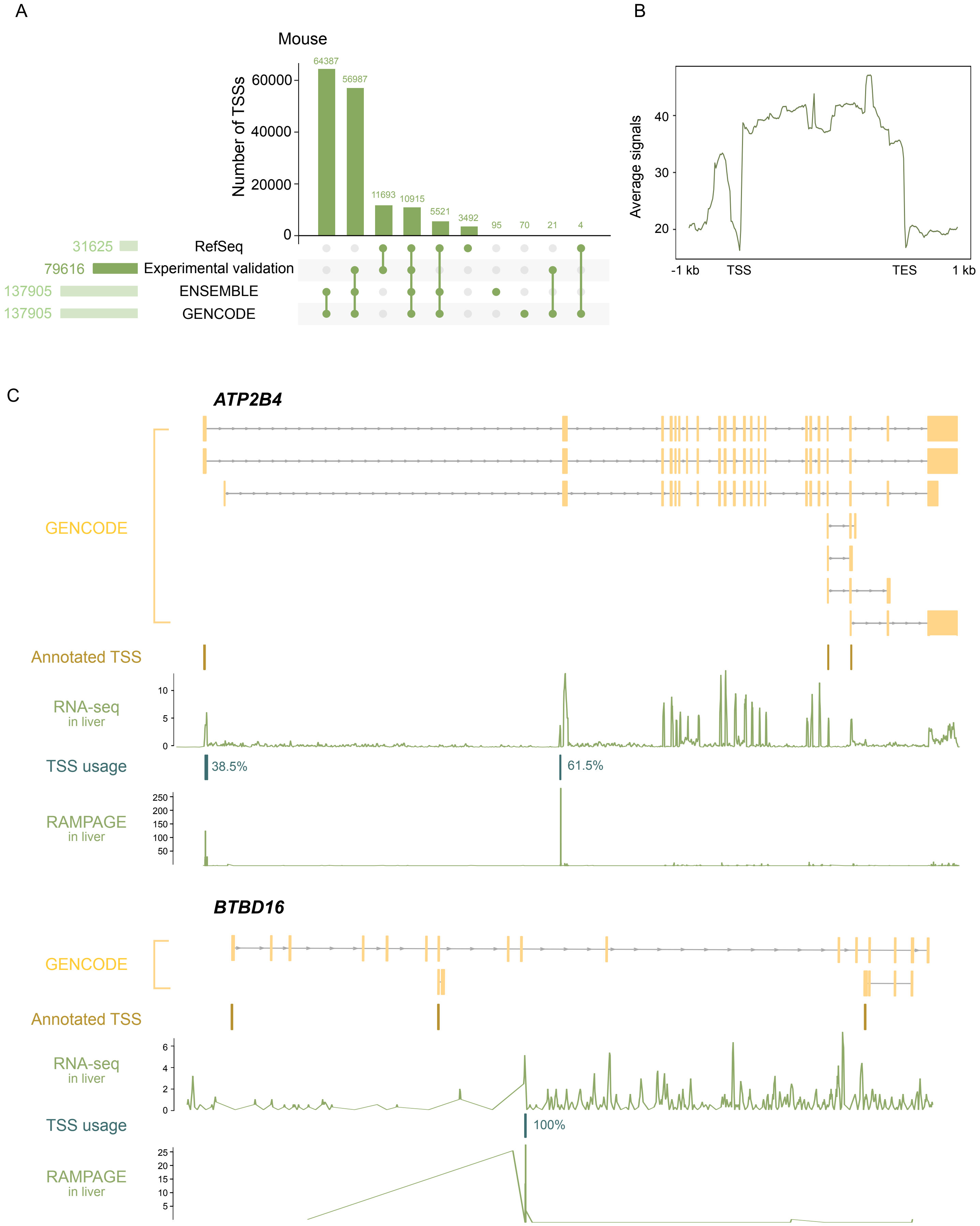
A
Mouse
Number of TSSs
60000
40000
20000
0
64387
56987
11693
10915
5521
3492
95
70
21
4
31625
79616
137905
137905
RefSeq
Experimental validation
ENSEMBLE
GENCODE
B
Average signals
40
30
20
-1 kb
TSS
TES
1 kb
C
ATP2B4
GENCODE
Annotated TSS
RNA-seq
in liver
10
5
0
TSS usage
38.5%
61.5%
RAMPAGE
in liver
250
200
150
100
50
BTBD16
GENCODE
Annotated TSS
RNA-seq
in liver
6
4
2
0
TSS usage
100%
RAMPAGE
in liver
25
20
15
10
5

Figure S2

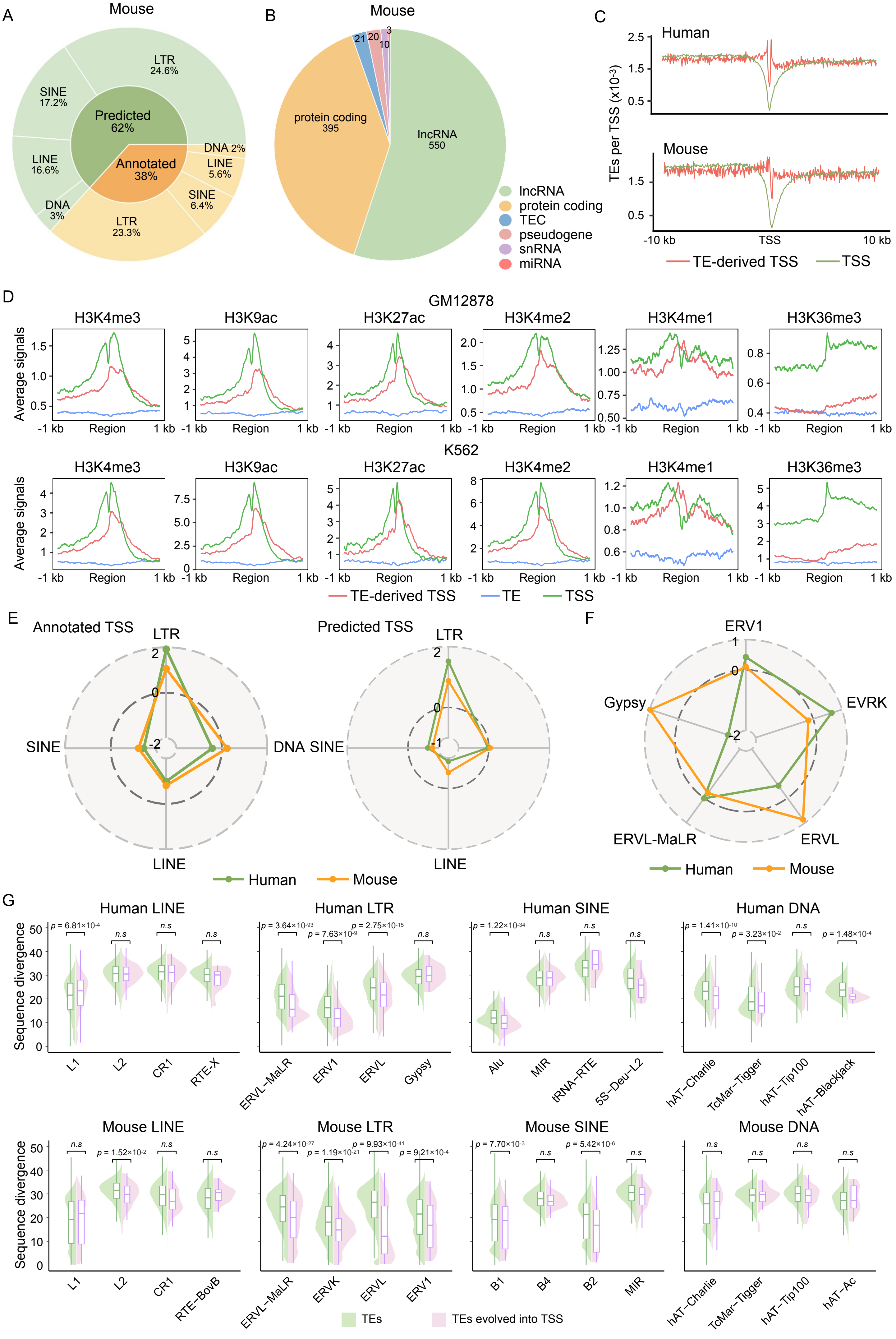
A
Mouse
Predicted
62%
Annotated
38%
LTR
24.6%
SINE
17.2%
LINE
16.6%
DNA
3%
DNA 2%
LINE
5.6%
SINE
6.4%
LTR
23.3%
B
Mouse
protein coding
395
lncRNA
550
21
20
3
10
lncRNA
protein coding
TEC
pseudogene
snRNA
miRNA
C
Human
Mouse
TEs per TSS (x10-3)
-10 kb
TSS
10 kb
TE-derived TSS
TSS
D
GM12878
K562
H3K4me3
H3K9ac
H3K27ac
H3K4me2
H3K4me1
H3K36me3
Average signals
-1 kb
Region
1 kb
TE-derived TSS
TE
TSS
E
Annotated TSS
Predicted TSS
LTR
SINE
DNA
LINE
Human
Mouse
F
ERV1
EVRK
ERVL
ERVL-MaLR
Gypsy
Human
Mouse
G
Human LINE
Human LTR
Human SINE
Human DNA
Mouse LINE
Mouse LTR
Mouse SINE
Mouse DNA
Sequence divergence
L1
L2
CR1
RTE-X
ERVL-MaLR
ERV1
ERVL
Gypsy
Alu
MIR
tRNA-RTE
5S-Deu-L2
hAT-Charlie
TcMar-Tigger
hAT-Tip100
hAT-Blackjack
RTE-BovB
ERVK
B1
B4
B2
hAT-Ac
n.s
TEs
TEs evolved into TSS

Figure S3

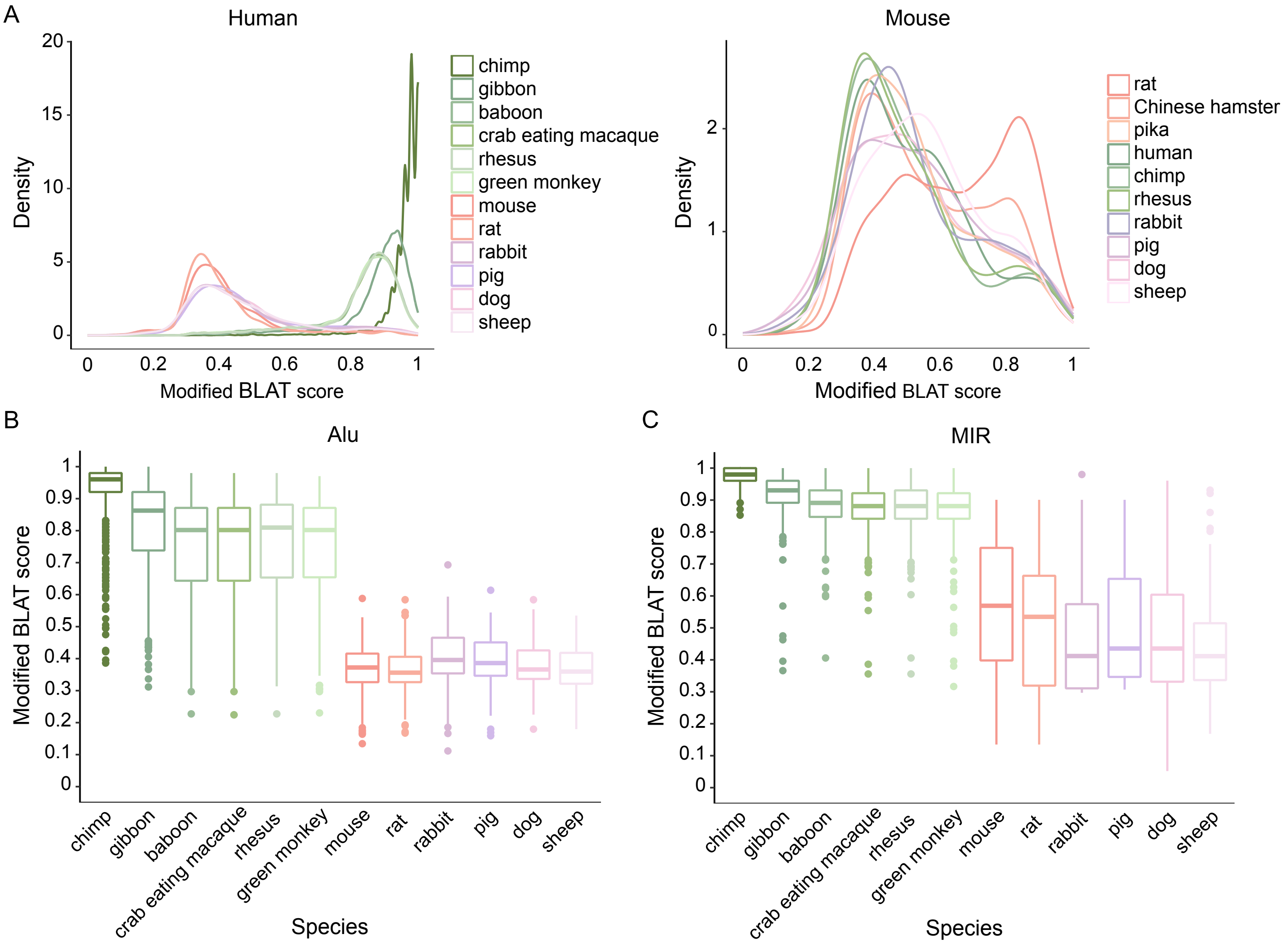
A
Human
Density
Modified BLAT score
chimp
gibbon
baboon
crab eating macaque
rhesus
green monkey
mouse
rat
rabbit
pig
dog
sheep
Mouse
Density
Modified BLAT score
rat
Chinese hamster
pika
human
chimp
rhesus
rabbit
pig
dog
sheep
B
Alu
Modified BLAT score
Species
chimp
gibbon
baboon
crab eating macaque
rhesus
green monkey
mouse
rat
rabbit
pig
dog
sheep
C
MIR
Modified BLAT score
Species
